# A scoping review of the Goldilocks mastectomy

**DOI:** 10.1016/j.jpra.2025.09.031

**Published:** 2025-10-05

**Authors:** Lauren L. van Tits, Olga F. Marin, Iris Holt-kedde, Vera C. van Aalst, Martinus M. van Veen

**Affiliations:** a University of Groningen, University Medical Center Groningen, Groningen, the Netherlands; b Department of Plastic Surgery, University Medical Center Groningen and University of Groningen, Groningen, the Netherlands

**Keywords:** Goldilocks mastectomy, Breast reconstruction, Autologous reconstruction, Oncoplastic surgery, QoL, Complication

## Abstract

**Background:**

The Goldilocks mastectomy was originally developed as a “middle ground” between a breast reconstruction and a modified radical mastectomy, particularly for obese women who are poor candidates for direct breast reconstruction. This scoping review aims to provide an overview of surgical techniques, complications, cosmetic outcomes and patient-reported quality of life following the Goldilocks mastectomy.

**Methods:**

Following the PRISMA-ScR guidelines a systematic search was conducted in March 2025, using PubMed and Embase. The identified studies were screened by two independent reviewers, based on predefined inclusion and exclusion criteria. Data focusing on surgical technique, complications and outcomes was extracted and recorded.

**Results:**

Fourteen articles were included, representing a total of 486 women (758 breasts). The majority were retrospective cohort studies. The Goldilocks mastectomy techniques reported varied, with some studies describing modifications to add more volume or cause less scarring. Common complications included seroma, wound dehiscence and fat necrosis. Four studies reported on secondary surgeries for volume enhancement. Three studies assessed cosmetic outcomes, which showed mixed results. Patient-reported quality of life, measured using the BREAST-Q, showed scores comparable to traditional reconstruction techniques.

**Conclusion:**

Goldilocks mastectomy provides a safe reconstructive option, especially for obese patients. Complication rates are comparable to those found in traditional mastectomy techniques and patient satisfaction appears favorable. However, limited high-quality evidence exists regarding long-term cosmetic outcomes and quality of life measurements. Further research using more standardized outcome measures is needed to fully evaluate the pros and cons of the Goldilocks mastectomy in breast reconstruction.

## Introduction

Breast cancer is the most commonly occurring cancer in women and an increasing number of these women subsequently opt for breast reconstruction. Traditionally, breast reconstruction procedures can be divided into autologous, free flap or pedicled flap (e.g., latissimus dorsi) surgery, or alloplastic procedures, using implants or tissue expanders. In obese patients, this poses a challenge in both instances due to a higher chance of complications.^1^

Goldilocks mastectomy offers a reconstruction technique for obese patients or patients with ptotic breasts that are poor candidates for other post mastectomy reconstructive options. In Goldilocks mastectomy, as originally defined by Richardson and Ma,^2^ a skin sparing mastectomy is initially performed using a peri-areolar approach, and then, using the wise-pattern incisions, the residual tissue is used to form a breast mound. Since its original introduction, many variations of the procedure have been introduced, and yet are all still referred to as Goldilocks mastectomy. Rather than just being used for obese patients or patients with ptotic breasts, the procedure is increasingly being used for breast reconstruction in women who prefer to save as much of their tissue as safely possible at the time of subcutaneous mastectomy.^3^ Although when choosing the Goldilocks procedure women must accept the small breast volume, they appreciate the option of preserving their nipple areola complex (NAC), as possible with the novel Goldilocks alternatives, and all the reconstructive options that are still available at a later stage.

The objective of this review is to analyze existing published information about Goldilocks mastectomy and provide a baseline for further studies focused on this procedure. This publication provides an overview of the Goldilocks mastectomy procedure: the operation technique (with variations), complications, cosmetic outcomes and quality of life assessments of patients subject to this intervention.

## Methods

This scoping review is reported in accordance with the Preferred Reporting Items for Systematic reviews and Meta-Analyses extension for Scoping Reviews (PRISMA-ScR) guidelines.^4^

### Search strategy

The search strategy was formulated collaboratively by all its authors. The systematic search was conducted in March 2025 using the bibliographic databases PubMed and Embase. Keywords included in the search strategy were “Goldilocks AND breast.” The keyword “Goldilocks” contains the name of the surgical procedure, and the term “breast” was added to clarify the article is about a form of mastectomy. No additional filters or limitations were applied to the search query to capture all relevant articles discussing the Goldilocks mastectomy procedure.

### Eligibility criteria

Eligibility criteria for this review were defined using the PICO framework (Table 1).^5^ A predefined set of inclusion and exclusion criteria was established by all authors to determine study eligibility for inclusion in the final review. Inclusion criteria included: (1) studies focusing on the Goldilocks mastectomy procedure; (2) studies written in English, Dutch, French, German or Spanish and (3) Original research articles presenting clinical data or surgical techniques.

**Table 1:** Eligibility criteria according to the PICO framework.

Acronym	Definition	Description
P	Population	Patients undergoing the Goldilocks mastectomy procedure
I	Intervention	The Goldilocks Mastectomy, with or without modification (e.g., variations in technique, alloplastic augmentation)
C	Comparison	Not applicable
O	Outcomes	Surgical techniques, complication rates, patient-reported results and aesthetic results related to the Goldilocks mastectomy

Exclusion criteria were: (1) studies not addressing the Goldilocks mastectomy; (2) single-patient case reports; (3) literature review articles; (4) invited commentaries or expert opinions; (5) conference abstracts and (6) articles without accessible full-text.

### Study selection and data extraction

Duplicates were removed from all articles identified by the search query, after which all remaining articles were independently screened by two authors (OM and LLvT) based on title and abstract. Any disagreement between the two reviewers on inclusion or exclusion of an article was resolved by automatically including the article in the subsequent screening phase. This second phase of screening involved full-text screening, also conducted independently by the two authors (OM and LLvT). In case of disagreement in the full texts, this particular article was discussed by all authors and consensus was reached. Cohen’s kappa was calculated to determine the interrater reliability of title and abstract screening and full-text screening.

Study characteristic data was extracted from the selected articles by two authors (OM and LLvT): country, publication year, study design, sample size, number of breasts, age, BMI, follow-up time, surgical technique, complications, patient reported outcomes and secondary surgeries.

### Quality assessment

To assess the methodological quality of the included articles, the validated version of the methodological index for non-randomized studies (MINORS) criteria was used.^6^ The methodological quality of the included articles was assessed independently by two authors (MMvV and LLvT) and consensus was reached by comparing all scores. Cohen’s kappa was calculated to determine the interrater reliability of the quality assessment. For this review, a total MINORS score of ≤8 was classified as poor quality, 9–14 as fair quality and 15–16 as excellent quality for non-comparative studies.^6^^,^^7^ The corresponding cut-off points for comparative studies were ≤14 for poor quality, 15–22 for fair quality and 23–24 for excellent quality.

## Results

**Figure 1:**
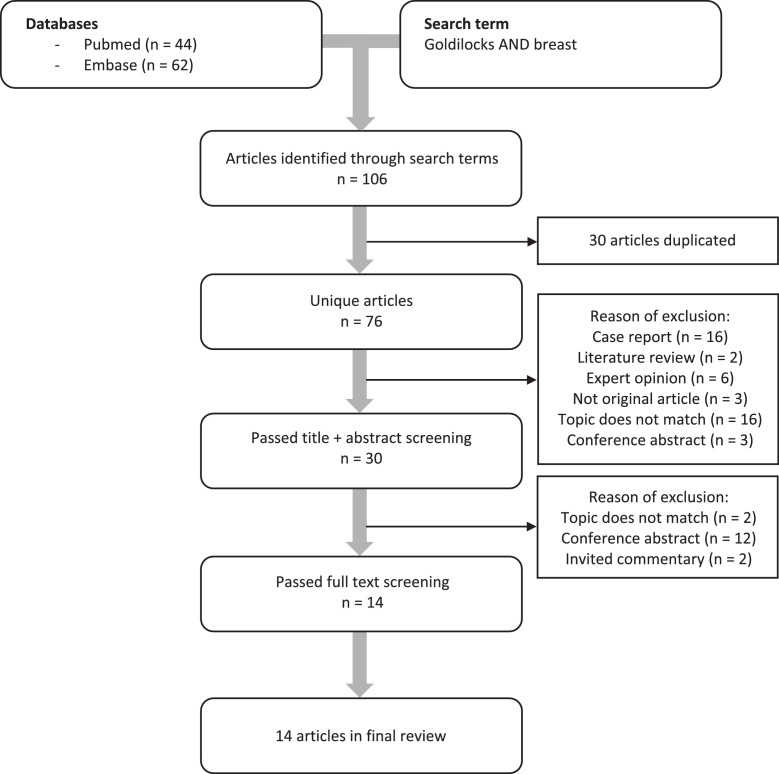
Flowchart following PRISMA guidelines of search strategy and study selection completed with reasons of exclusion.

The total number of articles identified was 106. After the exclusion of duplicates, a total of 76 articles remained and were included in the first screening stage. Following the title and abstract screening, 46 articles were excluded due to various reasons (Figure 1). Of the remaining 30 articles, a full-text screening was done, after which 14 articles met the inclusion and exclusion criteria.^2^^,^^3^^,^^8^, ^9^, ^10^, ^11^, ^12^, ^13^, ^14^, ^15^, ^16^, ^17^, ^18^, ^19^ Cohen's Kappa for title and abstract screening was 0.92 (absolute agreement 96 %). Cohen’s Kappa for full-text screening was 1.0 (absolute agreement 100 %).

### Results quality assessment

Table 2 presents scores for each article per item of the MINORS criteria. Cohen’s Kappa was 0.457 (absolute agreement 67.5 %). According to the MINORS scoring scale, seven articles were considered to be poor quality and seven articles fair quality.

**Table 2:** Articles scored on individual items of the MINORS criteria.

### Study characteristics

Of the 14 articles analyzed, nine were retrospective cohort studies, three were prospective cohort studies and two were case series. All papers were published between 2012 and 2025 and were conducted in multiple countries. A total of 486 patients and 758 breasts are presented, with a sample size ranging from two to 105 (Table 3).

**Table 3:** Study characteristics.

Author	Year	Country	Study design	Cohort	Amount of breasts	Age (Year)	BMI	Follow-up (months)
Alhussini et al. ^8^	2024	Egypt	Prospective cohort study	55	55	56 (50–62)^b^	41.2 (36.6–44.3)^b^	36 (28–45)^b^
Becker et al. ^9^	2021	USA	Retrospective chart review	3	6	69 (54–79)^a^	36 (30.2–40.8)^a^	11.3 (8–16)^c^
Binks et al.^10^	2024	Australia	Case series	2	4	66 ± 1.0	NR	6
Bustos et al.^11^	2021	USA	Retrospective cohort study	105	181	57.1 ± 1.0^e^ and 51.5 ± 1.1^f^	40.5 ± 0.7^e^ and 35.3 ± 0.4^f^	15.1 (10–28.6)^b^
Chaudhry et al.^12^	2019	USA	Retrospective cohort study	53	96	55.8 (33–77)^c^	33.7 (19.2–54.6)^c^	NR
Ghanouni et al.^13^	2023	USA	Retrospective cohort study	58	83	56 (34–78)^c^	36.8 (25.9–55)^c^	9 (1–54)^c^
Manrique et al.^14^	2022	USA	Retrospective cohort study	63	108	57.8 ± 10.1	37.6 ± 8.7	15 (6–52)^a^
Ogawa^15^	2015	Japan	Case series	5	6	72 (67–76)^c^	29.3 (25.1–39)^c^	NR
Richardson and Ma^2^	2012	USA	Retrospective cohort study	32	50	56 (41–94)^a^	30 (18–51.9)^a^	2–22^d^
Schwartz^16^	2017	USA	Retrospective cohort study	10	20	NR	45 (37–50)^c^	> 9
Schwartz^17^	2019	USA	Prospective cohort study	14	28	NR	33.5 (24.4–44)^c^	6
Setit et al.^3^	2023	Egypt	Prospective cohort study	15	18	51.6 (33–70)^c^	39.1 (31–46)^c^	15 (1–24)^a^
Wang et al.^18^	2024	USA	Retrospective cohort study	66	94	58 (33–84)^c^	NR	45 (1–54)^c^
Yee et al.^19^	2023	Singapore	Retrospective cohort study	5	9	NR	NR	NR

NR = Not reported.

a Reported as “median (range).”

b Reported as “median (interquartile range).”

c Reported as “mean (range).”

a Reported as “range.”

a Goldilocks-only group, 57 patients and 96 breasts.

f Goldilocks with implant-based immediate breast reconstruction, 48 patients and 85 breasts.

### Study outcomes

#### Patient group

The data included a total of 486 patients. BMI ranged from 19.2 to 54.6 with 10 out of 14 articles reporting a mean BMI > 30. Ages ranged from 33 to 94 years. A total of 214 women underwent a unilateral procedure, and 272 women received bilateral Goldilocks mastectomies. The two primary indications for surgery were a diagnosis of (unilateral or bilateral) breast cancer or prophylactic mastectomy for risk reduction. While the Goldilocks mastectomy is popular in obese patients, six articles included women with a BMI under 30 kg/m^2^.^2^^,^^12^, ^13^, ^14^, ^15^^,^^17^

#### Surgical techniques

While all procedures discussed in this paper were referred to as “Goldilocks,” the surgical techniques described in the different papers varied (Table 4).

**Table 4:** Surgery techniques and relevant additional procedures per article.

Article	Surgery technique	Nipple reconstruction	Additional surgeries
Alhussini et al., ^8^	Goldilocks mastectomy with advanced breast shaping	–	–
Becker et al.,^9^	Goldilocks mastectomy without a vertical incision	–	33 %
Binks et al.,^10^	–	Free nipple grafting	–
Bustos et al.,^11^	Originally Goldilocks mastectomy - with immediate breast reconstruction	Local tissue flaps	34.4 %–63.5 %
Chaudhry et al.,^12^	Originally Goldilocks mastectomy	–	–
Ghanouni et al.,^13^	Originally Goldilocks mastectomy	–	35 %
Manrique et al.,^14^	Originally Goldilocks mastectomy	Local tissue flaps	40,70 %
Ogawa,^15^	Originally Goldilocks mastectomy	–	–
Richardson and Ma,^2^	Originally Goldilocks mastectomy	Prosthetic silicone nipples	–
Schwartz,^16^	Goldilocks combined with second-stage reconstruction	Free nipple grafting	100 %
Schwartz,^17^	Goldilocks combined with LICAP flap	Free nipple grafting	–
Setit et al.,^3^	Nipple sparing Goldilocks mastectomy	NAC preservation	–
Wang et al.,^18^	Originally Goldilocks mastectomy	–	31,90 %
Yee et al.,^19^	Goldilocks combined with MCW-LICAP flap	–	–

In the original Goldilocks mastectomy, first described by Richardson and Ma,^2^ the Nipple areola complex (NAC) and glandular tissue of the breast were removed. For the remaining tissue a wise pattern was used to create an autologous breast mound. The caudal flap (skin and subcutaneous tissue) was de-epithelialized, providing some volume underneath the remaining skin envelope. This procedure resulted in an inverted T-shaped scar, with the horizontal component along the inframammary fold and the vertical component extending from the fold toward the nipple. This technique, as originally described was performed in seven of the 14 studies included herein, with a total of 315 patients (501 breasts).^2^^,^^11^, ^12^, ^13^, ^14^, ^15^^,^^18^

In one paper, by Becker et al. a variation of the Goldilocks mastectomy procedure is described in which no vertical incisions were made.^9^ Three patients (six breasts) underwent this variation. In contrast to the original Goldilocks mastectomy, this modification does not utilize wise-pattern incisions. Instead, a horizontal incision was made superior to the NAC, along with a second horizontal incision following the inframammary fold. The skin between these incisions was de-epithelialized, a breast mound was then created, and the breast was closed, resulting in a single horizontal scar along the inframammary fold.

Two other modifications of the original Goldilocks mastectomy included the use of lateral intercostal artery perforator (LICAP) flaps and modified chest wall LICAP (MCW-LICAP) flaps in combination with the original procedure.^17^^,^^19^ These techniques aimed to provide additional volume by recruiting adjacent tissue. Schwartz (2019)^17^ described a cohort of 14 patients (28 breasts), who underwent the Goldilocks procedure combined with LICAP flaps, resulting in a vertical scar extending to the nipple and a horizontal scar continuing towards the scapula. Yee et al.^19^ described five patients (nine breasts) who underwent the Goldilocks mastectomy in combination with MCW-LICAP flaps, where the incision of the lateral flap was adjusted by the natural tissue tension and lines.

Alhussini et al.^8^ described a modification of the Goldilocks procedure in 55 women (55 breasts), in which a more rounded breast contour was achieved, and more breast volume was preserved. One key difference from the original Goldilocks mastectomy procedure was the minimal excision of subcutaneous fat, which contributed to an increase in breast volume. Additionally, the use of a three-in-one suture and so-called “hypnotic sutures,” allowed for anchoring of the upper corners of the lower de-epithelialized flap and the sides of the breast mound to the chest wall. The authors reported that this approach enhanced a more rounded contour of the breast.

Bustos et al.^11^ described a modification of the original Goldilocks procedure, in which 48 patients (85 breasts) underwent an immediate breast reconstruction with alloplastic augmentation. In this case, silicone implants or tissue expanders were placed at the time of the Goldilocks mastectomy, to achieve volume restoration.

In six studies, nipple reconstruction was added to the Goldilocks procedure, utilizing various techniques.^3^^,^^10^^,^^11^^,^^14^^,^^16^^,^^17^ One article by Setit et al.^3^ describes a nipple-sparing Goldilocks mastectomy, in which the NAC is preserved by using a keyhole pattern. Three other articles describe a method of free nipple grafting simultaneously with the Goldilocks procedure^10^^,^^16^^,^^17^ and in two articles local flaps were used to reconstruct the nipple, which were performed either during the Goldilocks procedure or in a secondary procedure.^11^^,^^14^

Lastly, several studies reported on patients undergoing additional surgeries following their initial Goldilocks procedure. These included surgeries to increase volume using tissue expanders, implants, fat grafting or local and free flaps. Secondary surgeries following the Goldilocks procedure were reported in six articles, with 31.9 %–100 % of breasts undergoing at least one of these additional procedures,^9^^,^^11^^,^^13^^,^^14^^,^^16^^,^^18^ and three of the six articles reporting <40 %.^9^^,^^13^^,^^18^ Furthermore, Manrique et al.^14^ found that 45 % of the breasts that underwent a secondary surgery needed two or more additional surgeries. Schwartz (2017)^16^ focused on a two-stage procedure where the Goldilocks was followed by an implant-based reconstruction, and all breasts underwent secondary surgery. Bustos et al.^11^ highlighted the use of fat grafting as a key technique in secondary surgeries for volume enhancement following the Goldilocks procedure. Their findings indicated that 34.4 % of breasts that underwent an original Goldilocks mastectomy required one or more sessions of fat grafting, with a total of 342.7 mL ± 38.7 added volume for each breast.

#### Complications

Of the 14 studies included in this review, 10 reported postoperative complications associated with the Goldilocks mastectomy.^2^^,^^3^^,^^8^^,^^9^^,^^11^, ^12^, ^13^, ^14^^,^^17^^,^^18^ The most frequently reported complications were seroma, wound dehiscence and fat necrosis (Table 5). Nine studies^2^^,^^3^^,^^8^^,^^9^^,^^11^, ^12^, ^13^, ^14^^,^^18^ reported the occurrence of seroma, where the prevalence ranged from 2.1 %–16.7 % of breasts, and below 5 % in five out of nine studies. Wound dehiscence was reported in five studies with an occurrence ranging from 2.8 %–12.5 %.^3^^,^^8^^,^^11^^,^^12^^,^^14^ Five studies reported fat necrosis, where the occurrence ranged from 2 %–16 %.^2^^,^^8^^,^^14^^,^^17^^,^^18^ Other less frequent complications were reported and include hematoma, skin flap necrosis, infection and cellulitis. Five studies reported 1 %–16.7 % of reoperations, with the main indication being tissue necrosis.^9^^,^^11^, ^12^, ^13^^,^^18^

**Table 5:** Reported complication rate for diverse complications.

Article	Seroma (%)	Wound dehiscence (%)	Fat necrosis (%)	Hematoma (%)	Skin flap necrosis (%)	Infection (%)	Cellulitis (%)	Reoperation (%)
Alhussini et al.,^8^	5,5	3,6	9,1	3,6	–	–	–	–
Becker et al.,^9^	16,7	–	–	–	–	–	–	16,7
Binks et al.,^10^	–	–	–	–	–	–	–	–
Bustos et al.^11^^a^	7,3	12,5	–	1	4,2	6,3	–	4,2
Chaudhry et al.,^12^	2,1	3,1	–	1	–	–	2,1	1
Ghanouni et al.,^13^	4,8	–	–	6	1,2	4,8	–	7,2
Manrique et al.,^14^	4,6	2,8	15,7	0,9	0,9	–	–	–
Ogawa^15^	–	–	–	–	–	–	–	–
Richardson and Ma,^2^	2	–	2	–	–	–	6	–
Schwartz,^16^	–	–	–	–	–	–	–	–
Schwartz,^17^	–	–	14,3	–	–	–	–	–
Setit et al.,^3^	5,5	11	–	–	16,5	–	–	–
Wang et al.,^18^	4,3	–	16	2,1	2,1	4,3	–	6,4
Yee et al.,^19^	–	–	–	–	–	–	–	–

a Rates of “Goldilocks-only group” are reported.

#### Cosmetic outcomes

Three studies reported aesthetic outcomes after Goldilocks mastectomies.^3^^,^^8^^,^^15^ Setit et al.^3^ used a five-point scale to evaluate cosmetic outcomes, based on ratings by a panel of three surgeons. They reported 40 % (seven patients) that scored excellent, 22 % (four patients) good, 16 % (three patients) satisfactory, 11 % (two patients) poor and 11 % (two patients) very poor. Ogawa^15^ did not specify the method used to evaluate their aesthetic outcomes; however, the author reported one case had a good cosmetic result, based on the symmetry of the two breasts, and three cases that had poor cosmetic results due to size difference between the operated and the preserved breasts. Patient reported satisfaction with cosmetic outcomes using the Likert scale was reported by Alhussini et al.^8^ According to this study, 29.1 % were satisfied, 69.1 % were extremely satisfied and 1.8 % were unsatisfied.

#### Quality of life

Patient-reported quality of life after undergoing the original Goldilocks mastectomy was reported in two studies, both using the BREAST-Q survey.^11^^,^^14^ Results were presented in scores ranging from 0 to 100, with higher scores indicating higher levels of satisfaction. Manrique et al.^14^ reported median scores of 65 (range 30–92) for the domain satisfaction with breasts, 80 (range 20–100) for physical well-being, 80 (range 55–100) for psychosocial well-being and 68 (range 31–200) for sexual well-being. Bustos et al.^11^ reported median scores of 83.3 (interquartile range 66.7–91.7) for satisfaction with breasts, 96.7 (interquartile range 81.7–100) for physical well-being, 86 (interquartile range 78.0–96.0) for psychosocial well-being and 52 (interquartile range 40.0–81.7) for sexual well-being.

## Discussion

The aim of this review was to make an overview of the published information reported in the literature about the Goldilocks mastectomy procedure to identify any potential knowledge gaps and further implications of the technique.

Although BMI is a known risk factor for complications in breast surgery, the complication rates reported in the articles we reviewed are similar to traditional mastectomy procedures and well within the range of complications known for autologous (±40 %) and alloplastic (±25 %) breast reconstructions.^20^ Furthermore, the most common complications were seroma and wound dehiscence, both relatively easy to manage. Tissue necrosis was less common and is relatively easily managed if no implants are placed at the time of surgery. This review suggests that the Goldilocks mastectomy is a safe alternative in overweight women undergoing a mastectomy.

Only two out of the 14 studies included in our review reported on quality of life, both using the BREAST-Q questionnaire.^11^^,^^14^ The scores reported are similar to those of women undergoing alloplastic or autologous breast reconstruction.^21^ There is also a significant difference in scores between Goldilocks mastectomies and a traditional mastectomies, especially in the domain of physical well-being, leading to the idea that Goldilocks mastectomy results in a better quality of life.^22^

Three studies reported cosmetic outcomes, using two different methods.^3^^,^^8^^,^^15^ Two of the studies used a five-point scale and reported mainly good and satisfactory outcomes (surgeon panel vs patient reported), while the other used an unspecified assessment method and reported mainly poor outcomes. This discrepancy could be due to the variability in methods or from the very small sample size in the study by Ogawa.^15^ Nevertheless, the Goldilocks mastectomy procedure offers both a mastectomy and a partial reconstruction, as opposed to a mastectomy alone. Along these lines, one could argue that the cosmetic outcomes, even though not that desired by the patient, is better than only that of a traditional mastectomy. Moreover, Goldilocks mastectomy allow nipple-areolar complex sparing surgery, which could also improve cosmetic outcomes and patient satisfaction.

While the Goldilocks technique was initially introduced for patients unsuitable for more extensive reconstruction, recent refinements suggest broader applicability. Women with macromastia or ptotic breasts, including those who are overweight, may benefit from this technique as a primary option rather than a fallback. The refined version of the procedure allows for NAC preservation and improved breast shaping, making it more appealing for patients seeking aesthetic enhancement. Its ability to provide a breast mound without donor site morbidity, foreign material, or multiple procedures makes it especially attractive for high-risk patients desiring a single-stage, less invasive approach.

Some authors have described adaptations involving pedicled flaps or implants to enhance volume. While these may improve outcomes in selected cases, they introduce additional surgical complexity and morbidity, which must be weighed against the original minimally invasive intent of the procedure. On the other hand, even in its refined form, the Goldilocks technique may not always meet patients' aesthetic expectations, particularly in terms of volume and projection, which are generally less than with flap-based reconstruction.

Currently, direct comparison with other reconstructive techniques remains limited due to selection bias as the Goldilocks technique is most often reserved for patients not eligible for other options. Broader application of the technique may, in the future, allow for more balanced and meaningful comparative studies.

A variation from the original Goldilocks procedure is its indication in women with a normal BMI. For these patients the Goldilocks procedure (sparing the nipple areola complex, skin and subcutaneous fat) may offer an ideal choice at the time of the oncological or risk reducing procedure. Compared to lumpectomy, radiation therapy is much less frequently needed. If the remaining breast volume is unsatisfactory, all options to increase breast volume will still be available. We therefore believe that a plastic surgeon should include the Goldilocks procedure when informing patients about all the options for breast reconstruction!

### Strengths and limitations

In this review, we provide an overview of the studies in the literature using the Goldilocks mastectomy procedure, as it was originally described by Richardson and Ma^2^ as well as all the variations of the procedure since its introduction. We also describe complications, cosmetic outcomes and quality of life measurements reported in the literature.

Limitations of this review were the limited number of studies available in the literature and the fact that most of the studies analyzed were retrospective, and not prospective. In terms of parameters, not many studies focused on the subjective parameters, and for one of the two mentioned, “cosmetic outcomes,” a lack of standardized measures was a limitation, making it difficult to compare findings side by side.

### Implications

The Goldilocks mastectomy was first described as the “middle ground” between formal reconstruction after mastectomy and the amputated appearance associated with mastectomy without reconstruction. This review found that the technique is currently employed primarily in overweight women undergoing breast surgery, in cases where an extensive reconstruction is associated with too much risk. In these patients, Goldilocks mastectomy has a relatively low complication rate and a high level of patient satisfaction. Future studies are needed for a better understanding of the full potential of this technique. More studies including standardized patient-reported outcomes are necessary. Furthermore, Goldilocks mastectomies provide an option to preserve a greater amount of tissue and the nipple areola complex, without having to decide on a definitive breast reconstruction approach at the time of oncological surgery. This alternative may also be of interest for non-overweight women suffering from breast cancer.

## Conclusion

In conclusion, Goldilocks mastectomy represents a promising surgical technique for obese breast cancer patients. It is a curative treatment and provides immediate partial reconstruction of the breast, which may lead to higher satisfaction than a traditional mastectomy without increasing complication rates. Our literature review indicates that there is a lack of standardization of the technique, as more variations of the operation are cumulatively named “Goldilocks” and more research is needed on patient reported outcomes such as quality of life and cosmetic outcomes, as the patient groups are small and the methods used vary between studies.

## Funding statement

This research did not receive any specific grant from funding agencies in the public, commercial, or not-for-profit sectors. The article processing charge was covered by the University Medical Center Groningen (UMCG).

## Ethical approval statement

Ethical approval was not required for this study.

## Declaration of competing interest

The authors declare that they have no conflict of interest related to this study.
